# Draft Genome Sequence of *Streptomyces silvensis* ATCC 53525, a Producer of Novel Hormone Antagonists

**DOI:** 10.1128/genomeA.00001-16

**Published:** 2016-02-18

**Authors:** Chad W. Johnston, Yongchang Li, Nathan A. Magarvey

**Affiliations:** a Department of Biochemistry and Biomedical Sciences, McMaster University, Hamilton, Ontario, Canada; b Department of Chemistry and Chemical Biology, McMaster University, Hamilton, Ontario, Canada

## Abstract

*Streptomyces silvensis* produces nonribosomal peptides that act as antagonists of the human oxytocin and vasopressin receptors. Here, we present the genome sequence of *S. silvensis* ATCC 53525 and demonstrate that this organism possesses a number of additional biosynthetic gene clusters and might be a promising source for genome-guided drug discovery efforts.

## GENOME ANNOUNCEMENT

*Streptomyces* is a genus of *Actinobacteria*, and its members are known as some of the most prolific producers of polyketide and nonribosomal peptide natural products. *Streptomyces silvensis* was originally isolated by researchers at Merck, Sharp, and Dohme and was found to produce a series of cyclic peptides that acted as antagonists of oxytocin and vasopressin-human peptide hormones that control physiological processes, including contractions of uterine and mammary tissues (1). In hopes of identifying a candidate gene cluster for these valuable molecules, and to assess the biosynthetic potential of this industrially valuable bacterium, we isolated genomic DNA and performed whole-genome sequencing.

Genomic DNA was isolated using a previously described approach for Gram-positive *Actinobacteria*, such as *Streptomyces* (2). Sequencing was performed on an Illumina MiSeq DNA sequencer at the Farncombe Metagenomics Facility at McMaster University, Hamilton, Ontario, Canada. Library preparation used the Nextera XT sample preparation kit (Illumina). This library was sequenced using a 2 × 250-bp version 2 reagent kit, providing roughly 4.7 million reads and sequencing coverage of 53×. Raw reads were assembled into contigs using the ABySS assembler (3). The draft genome was found to possess a G+C content of 72.1% and contained 9,741,331 nucleotides. Annotation with Glimmer demonstrated that of the 92 contigs obtained from our assembly, 48 contigs contained 7,593 putative protein-coding genes.

Analysis of this genome with PRISM (4) identified 29 modular natural product biosynthetic gene clusters, including 20 clusters for nonribosomal peptide genes, 3 clusters for polyketides, and 6 clusters for hybrid peptides-polyketides. Among these, we identified a putative nonribosomal peptide gene cluster responsible for the production of the oxytocin antagonist cyclic peptides. We also found gene clusters responsible for the production of faeriefungin and echinomycin (5), which we had previously identified as natural products produced by *S. silvensis*. In addition to these established products of *S. silvensis*, we also observed gene clusters with high homology to those for production of the known molecules prodigiosin, coelichelin, and griseobactin. Interestingly, we also identified two gene clusters for producing lipocyclocarbamate (6) and pyrrolizidine (7) nonribosomal peptides, which possess a conserved acyl dipeptide core modified by a unique monooxygenase (6, 7).

*Streptomyces* is an industrially important genus that has been a crucial component of natural product drug discovery platforms, producing a wide variety of antibacterials, antifungals, anticancer agents, and immunosuppressants. Here, we used genome sequencing to study *S. silvensis*, which produces a unique series of cyclic peptides that act as antagonists of pharmaceutically relevant hormones, and we found that this valuable bacterium possesses many peptide and polyketide gene clusters for molecules we have yet to discover.

### Nucleotide sequence accession numbers.

This whole-genome shotgun sequencing project has been deposited at DDBJ/EMBL/GenBank under the accession no. LOCL00000000. The version described here is the first version, LOCL01000000.
